# Benchmarking antibody clustering methods using sequence, structural, and machine learning similarity measures for antibody discovery applications

**DOI:** 10.3389/fmolb.2024.1352508

**Published:** 2024-03-28

**Authors:** Dawid Chomicz, Jarosław Kończak, Sonia Wróbel, Tadeusz Satława, Paweł Dudzic, Bartosz Janusz, Mateusz Tarkowski, Piotr Deszyński, Tomasz Gawłowski, Anna Kostyn, Marek Orłowski, Tomasz Klaus, Lukas Schulte, Kyle Martin, Stephen R. Comeau, Konrad Krawczyk

**Affiliations:** ^1^ NaturalAntibody, Szczecin, West Pomeranian, Poland; ^2^ Pure Biologics, Wrocław, Poland; ^3^ Department of Biochemistry, Molecular Biology and Biotechnology, Faculty of Chemistry, Wrocław University of Science and Technology, Wrocław, Poland; ^4^ Global Computational Biology & Digital Sciences, Boehringer Ingelheim Pharma GmbH & Co. KG, Biberach, Germany; ^5^ Biotherapeutics Discovery, Boehringer Ingelheim, Biberach, Germany

**Keywords:** drug discovery, antibodies, machine learning, biologics and biosimilars, clustering, language models (LMs)

## Abstract

Antibodies are proteins produced by our immune system that have been harnessed as biotherapeutics. The discovery of antibody-based therapeutics relies on analyzing large volumes of diverse sequences coming from phage display or animal immunizations. Identification of suitable therapeutic candidates is achieved by grouping the sequences by their similarity and subsequent selection of a diverse set of antibodies for further tests. Such groupings are typically created using sequence-similarity measures alone. Maximizing diversity in selected candidates is crucial to reducing the number of tests of molecules with near-identical properties. With the advances in structural modeling and machine learning, antibodies can now be grouped across other diversity dimensions, such as predicted paratopes or three-dimensional structures. Here we benchmarked antibody grouping methods using clonotype, sequence, paratope prediction, structure prediction, and embedding information. The results were benchmarked on two tasks: binder detection and epitope mapping. We demonstrate that on binder detection no method appears to outperform the others, while on epitope mapping, clonotype, paratope, and embedding clusterings are top performers. Most importantly, all the methods propose orthogonal groupings, offering more diverse pools of candidates when using multiple methods than any single method alone. To facilitate exploring the diversity of antibodies using different methods, we have created an online tool-CLAP-available at (clap.naturalantibody.com) that allows users to group, contrast, and visualize antibodies using the different grouping methods.

## Introduction

The development of antibody therapeutics relies on the identification of a suitable binder towards a clinically relevant target. Though computational methods promising fully *de novo* design are making advances (Wilman et al., 2022), well-established experimental protocols still dominate antibody discovery (Lu et al., 2020).

Therapeutic antibodies are primarily discovered via phage display or animal immunizations. These protocols produce a large number of potential binders in response to a target. In animal immunization, one would typically look for expanded clones upon antigen challenge (Saggy et al., 2012; Laustsen et al., 2021). In phage display, one would likewise focus on an ‘enriched’ set of sequences (Chan et al., 2014; Saka et al., 2021; Zhang, 2023) between rounds of panning. Antibodies in the original set can represent diverse epitopes and developability profiles. One is tasked to downsample the initial set of antibodies (1000s) coming from such experiments to a smaller set (10s). The smaller set is subjected to costlier, more detailed assays designed to further narrow the scope of drug lead candidates. Ideally, the diversity of representatives should offer a good balance between binding propensity, epitope bins, and developability profiles. Testing a set of functionally promising, but close-to-identical, molecules would yield close-to-identical assay results, not hedging the bets on covering a wide spectrum of functionalities and developability profiles. Randomly picking from the initial set of antibodies does not guarantee to select the ‘best’ or most diverse candidates, especially if there is a bias towards a set of clones.

Downsampling a diverse set of representatives was typically achieved by grouping the initial set of sequences and selecting representatives from these. For instance, in immunization, the ‘expanded clones’ were identified by grouping sequences by their V-J gene assignments, CDR-H3 lengths, and a high cutoff (>80% sequence identity). The so-called ‘clonotyping’ (Briney et al., 2016; Pelissier et al., 2022) has proven to be an accurate method, identifying not only expanded clones but also showing convergent development of antibodies across different individuals (Trück et al., 2015; Galson et al., 2020).

The drawback of clonotyping is that, though two clones could be different in sequence, they might still represent a similar binding mode, reducing the diversity of the down-sample. Therefore, methods looking at other diversity dimensions of antibody were introduced. These are grouping based on paratope (Richardson et al., 2021), structure (Krawczyk et al., 2018; Robinson et al., 2021; Spoendlin et al., 2023), or embeddings (Friedensohn et al., 2020). Such alternative grouping methods aim to improve upon the original clonotype method by selecting clones that would not be identified by sequence methods alone, increasing the diversity of the down-samples.

Paratope-based grouping calculates the similarity between any two antibodies from their predicted epitopes. It was shown that paratope predictions can be obtained from sequence alone, paradoxically, in the absence of the antigen (Liberis et al., 2018). Though paratope-based prediction does not outperform clonotyping, it offers more orthogonal picks than clonotype alone (Richardson et al., 2021), significantly increasing the diversity of the sample along the paratope-diversity dimension.

Paratope prediction implicitly takes structural information into account, so it was also proposed that the entire 3D conformation difference be used as a similarity measure for antibodies (Wong et al., 2021). Antibody structural models can be computed quite fast (within milliseconds for some deep learning methods these days (Jaszczyszyn et al., 2023)), making it possible to produce and compare large three-dimensional datasets. Early attempts at structural grouping involved shared templates for structural modeling or canonical classes (Krawczyk et al., 2018; Kovaltsuk et al., 2020). Later approaches started to take the entire structure of the antibody into account, proving utility in epitope binning (Robinson et al., 2021; Spoendlin et al., 2023). Similarly to paratope-based grouping, structure-based approaches do not outperform clonotyping, but provide alternative picks along the structural dimension, diversifying the down-sample.

A solution that implicitly tackles the multi-dimensional nature of antibodies is to encode them in a latent space. By allowing the neural network to devise the vectorized representation based on self-supervised learning (Rives et al., 2021; Brandes et al., 2022; Elnaggar et al., 2022; Lin et al., 2023), one can implicitly capture similarity measures such as same lengths and amino acid distributions but also some relationships that are not obvious (Wilman et al., 2022). Such latent representations using antibody-specific AntiBERTa were shown to reflect gene annotations (Leem et al., 2022). AntiBERTy was used to reveal mutational trajectories within repertoires (Ruffolo et al., 2021). Such an approach was taken by Friedensohn et al. wherein a machine learning model was devised to encode the CDR combinations (Friedensohn et al., 2020). In particular, authors employed the Variational Autoencoder (VAE) to predict cluster assignment, which led them to discover RSV-F binders that would not be captured by clonotype alone.

An approach combining the structure and machine learning approaches was proposed recently in the form of SurfaceID (Riahi et al., 2023). Here, the structural surface is divided into a triangular mesh with each vertex having physicochemical annotations of close-by atoms. The authors trained the embedding on the surface patches to be similar to the overlapping ones on the surface and dissimilar to the ones farther away. This scheme was used to cluster paratope and epitope residues simultaneously, which grouped the complexes by their antigen structures, and in some cases sub-groups based on different paratopes against the same epitope (Riahi et al., 2023).

Though, as described above, there are multiple similarity measures available for antibodies, it is not entirely clear what the benefits of one over another are, if any, in the absence of uniform benchmarking. Previous studies comparing clonotyping *versus* paratope clustering and clonotyping *versus* structural clustering concluded that the more sophisticated methods are orthogonal rather than outright better than the simplistic sequence-based methods (Richardson et al., 2021; Spoendlin et al., 2023). To the best of our knowledge, there was no head-to-head benchmarking of the methods available. To address this issue, here we benchmarked the five similarity measures currently available to antibodies: sequence (Li and GodzikCd-hit, 2006; Steinegger and Söding, 2017), clonotyping (Hershberg et al., 2015), paratope-based (Richardson et al., 2021), structure-based (Krawczyk et al., 2018), and embedding-based clusterings (Friedensohn et al., 2020). We benchmarked these on four datasets across binder prediction and epitope mapping. To facilitate employing conclusions of this work, we make an online application available, CLAP (clap.naturalantibody.com), enabling users to compare small sets of antibody sequences.

## Materials and methods

### Datasets used for benchmarking

Five datasets were employed for benchmarking antibody clustering methods (Table 1). The PTx dataset consisted of 1,113 sequences checked for binding against Pertussis Toxoid (PTx). In total, the dataset contained 363 binding and 749 non-binding paired heavy-light chains. The OVA dataset consisted of lineages that were tested for binding against ovalbumin (OVA) antigen. In total, the dataset contained 723 binding and 1,646 non-binding paired heavy-light chains. The dataset generated by Cao et al. was employed for epitope binning. It contains 3,051 antibody sequences divided into 12 epitope groups. These three datasets were employed to gauge the benefits of different similarity measures on different datasets and to see whether there exists a generalizable parametrization that provides satisfying results.

**TABLE 1:** Datasets used in this study.

Dataset	Number of sequences	Binders/Nonbinders	Purpose	Source
PTx	1,112	363/749	Classification of binder/non-binder	Richardson et al. (2021)
OVA	2,369	723/1,646	Classification of binding lineages	Goldstein et al. (2019)
Pure_Target1	76 + 16 (blind test)	41/35	Classification of binder/non-binder	Pure Biologics
Pure_Target2	76 + 94 (blind test)	35/41	Classification of binder/non-binder	Pure Biologics
Cao	3,051	Not applicable	Epitope binning	Cao et al. (2023)

As a blind test set for parameterizations, we have employed a binder/non-binder dataset contributed by Pure Biologics. The dataset consisted of antibodies for two targets, which we denote as Target1 and Target2. The antibodies for both targets were divided into two sets, first with known binding properties, second with antibodies that were unknown to bind/not-bind. The set with known binders served to study the optimal parametrization of the model and to check if it achieves equally optimal performance on the blind test. In total, Pure_Target1 had 41 binders and 35 non-binders in the known set and 16 in the blind set. Pure_Target2 had 35 binders and 41 non-binders in the known set and 94 sequences in the blind set. The datasets were supposed to be a realistic case of a dataset employed in antibody discovery, used to reveal the pitfalls and benefits of the approaches we employ.

A single-cell (paired VH/VL) PTx dataset was created based on material from five genetically modified mice carrying all of the human immunoglobulin variable region genes. Pertussis Toxoid (PTx) was used to immunize mice. Heavy chains from sorted splenic B cells from the same five animal models as the single cell paired data set were sequenced (using standard protocols). PTx-binding and non-binding labels were applied to the sequences by the use of surface plasmon resonance (SPR) and homogeneous time-resolved fluorescence (HTRF).

OVA dataset authors employed high-throughput single cell B-cell receptor sequencing (scBCR-seq) to obtain accurately paired full-length variable regions in a massively parallel manner. More than 250,000 B cells from rat, mouse, and human repertoires were sequenced to characterize their lineages and expansion. Furthermore, rats were immunized with chicken ovalbumin (OVA), and antigen-reactive B cells from lymph nodes of immunized animals were profiled. Ninety-three clones from the identified lineages were synthesized, expressed, and tested, and clones that were antigen-reactive were identified.

Sequences in the datasets Pure_Target1 and Pure_Target2 were derived from antibody discovery programs at Pure Biologics. The sequences used as the training set came from a standard screening process–they were picked from an antibody phage library panned on target cells, sequenced using Sanger method, expressed, and classified as binder/non-binder using flow cytometry. The sequences in blind/test set were derived from massive Pac-Bio long read sequencing of the panned library followed by sequence abundance analysis. The blind/test sets comprised sequences that were not discovered in the standard screening. After the *in silico* clustering process, all molecules from the blind/test set were expressed and their binding was evaluated in flow cytometry.

The Cao dataset was constructed on data from monoclonal antibodies isolated from individuals who had SARS-CoV-2 Omicron BA.2 and BA.5 breakthrough infections. High-throughput sequencing protocol and deep mutational scanning (DMS) platform were used to generate data.

Each dataset underwent an assessment for sequence diversity across samples, which was quantified through pairwise sequence identity calculations using Levenshtein Distance applied to residues from specific regions. The provided figures (Supplementary Figure S1) visually depict the distinctions among the datasets. Our analysis indicates that PTx, OVA, and CAO exhibit a more diverse distribution of sequences, contrasting with the datasets from Pure Biologics, which manifest lower diversity. Notably, across all datasets, the complementarity-determining regions (CDR) demonstrate greater diversity compared to the full sequences, as evidenced by their lower mean sequence identity.

### Similarity measures and grouping methods implemented and benchmarked

We employed five clustering methods, each employing its own similarity measure, for this study, given in Table 2. We engaged methods for benchmarking by selecting the commonly used ones (e.g., clonotyping and sequence clustering) as well as those that were proposed as providing a benefit with respect to the established methods (paratope, structure, and embedding). Each method comes with a range of modifiable parameters, given in Table 2. Length stratification indicates whether sequences can be grouped by the length of a sub-region, typically CDR-H3. Clustering target indicates on which region the similarity within a group will be considered–e.g., l3_h3 will only calculate the sequence identity on the combination of CDR-L3 and CDR-H3, not taking the rest of the sequence into account. It must be noted that throughout the manuscript we refer to ‘similarity measures’, which might introduce confusion with respect to commonly employed distinction between sequence similarity and identity. For sequences, we employ identity as a metric with ‘similarity’ being used as an umbrella term for the different measures to discern differences between antibodies.

**TABLE 2:** Grouping methods and parametrizations used in this study.

Method	Length stratification	Clustering target (what gets clustered)	Method-specific parameters
Clonotyping	None, cdrh3	cdrh3, l3_h3, heavy chain, light chain	Clonotype: [v, j], [v], Sequence identity threshold
Sequence clustering	None, cdrh3	cdrh3, l3_h3, heavy chain, light chain, cdrl3	Sequence identity threshold
Paratope clustering	None, cdrh3	Predicted paratope	Paratope identity threshold
Structure clustering	None, cdrh3, cdrh, cdrs_all	cdrs_all, cdrh3, l3_h3	Distance threshold (Å), For PTx structures prediction model: AB2 vs. NanoNet
Embedding clustering	For heavy + light: None, cdrh3, all_cdr For heavy-only: None, cdrh, cdrh3	For heavy + light: all, cdrs_all, l3_h3, cdrh3 For heavy-only: all, cdrh3, cdrhs_all	Embedding dimension: 144 vs. 768 Transformer type (768 dimension): heavy-only vs. heavy + light

“cdr” - complementarity-determining regions numbered by IMGT, “l3_h3” - residues selected from CDR-H3 and CDR-L3 IMGT regions, “cdrh” - residues selected from all cdr IMGT regions of heavy chain, and “cdrs_all” - residues selected from all cdr IMGT regions.

Method-specific parameters show features not falling into the two previous categories, such as the choice of 3D modeling method in case of structural clustering. Each of the clustering methods is briefly described below.

#### Clonotyping

Antibody sequences are grouped by their assigned variable (V) region genes and CDR-H3 lengths. Such groups are then further divided by CDR-H3 sequence identity with cutoffs such as 70% or 80%. The definition of clonotype varies depending on the study, as gene assignments or the definition of the CDR-H3 could be distinct. For the purposes of this study, we benchmarked gene grouping by either V or V + J (joining) regions.

#### Sequence clustering

In practical terms, sequence clustering is cognate to clonotyping, in that sequences are grouped by their sequence identity as given by MMseqs2 (Steinegger and Söding, 2017). The method-specific parameter employed here is the sequence identity threshold calculated on the specific region of the aligned sequence (e.g., entire variable region, only the CDR-H3, *etc.*).

#### Paratope clustering

This involves grouping the sequences by their predicted paratopes. The method takes advantage of surprisingly good paratope prediction using deep learning in the absence of antigen (Liberis et al., 2018). The sequence identity is only calculated on the residues annotated as paratope, according to a threshold. Here, we developed a paratope prediction based on a language model following the protocol described by AntiBERTa (Leem et al., 2022).

#### Structure clustering

Antibodies are grouped by their structural similarity as calculated by root-mean-square deviation (RMSD) of C-alpha atoms of a select region (e.g., CDR-H3). Structural clustering is composed of two algorithms: the modeling step and the clustering step. For the modeling step, we benchmarked ABodyBuilder2 (Abanades et al., 2023), a freely-available antibody-specific adaptation of AlphaFold2, which takes several seconds per prediction, and a reconstruction of NanoNet, without the benefit of structural pre-training (Kończak et al., 2022). The latter network is the simplest form of the machine learning antibody structure predictor that we are aware of, whereas AB2 represents the state of the art in the form of AlphaFold2 (Jaszczyszyn et al., 2023). Contrasting both approaches was designed to reveal whether using slower, more sophisticated methods has a benefit over the simpler, faster ones. Since the similarity between the antibodies is calculated as RMSD, it is a computationally expensive calculation. For this reason, we benchmarked mTM-Align (Dong et al., 2018a) which performs all-vs-all structural alignment, and SPACE (Robinson et al., 2021; Spoendlin et al., 2023) that introduces a greedy algorithm, reducing the number of calculations.

#### Embedding clustering

Antibody sequences can be vectorized into more efficient representation (embedding) in latent space learned by self-supervised methods (Wilman et al., 2022). We employed the BERT architecture, with the Masked Language Modelling (MLM) objective that was successfully used in general proteins (Rives et al., 2021; Lin et al., 2023) and subsequently in AntiBERTa (Leem et al., 2022) and AntiBERTy (Ruffolo et al., 2021). We checked two sizes of transformers: small, with an embedding size of 144, and large, with an embedding size of 768. Further to that, we checked two variations of transformers, namely, paired and unpaired (Burbach and Briney, 2023). The paired transformers were trained on 1.3 million paired sequences from a recent study by Jaffe et al. (Jaffe et al., 2022). The unpaired transformer was trained on 23 million high-quality sequences from our cleaned, corrected, and updated version of the Observed Antibody Space (OAS) dataset (Kovaltsuk et al., 2018). The embeddings were taken as the last layer.

### Accuracy metrics

#### Binding prediction metrics

The PTx, OVA, and Pure Biologics datasets consist of binders and non-binders. The only exception here is that the OVA dataset consists of lineages where binders were detected. In each case, though, we assume that we have two disjoint sets of antibody binders and non-binders. Subsequently, we employ a probe-mining approach (Richardson et al., 2021), wherein we select one known binder and occlude the label of all other sequences. The sequences that cluster with the probe are marked as ‘binders’, all others as non-binders. On this basis, we can calculate the precision, recall, and derivative metrics for all grouping methods.

#### Epitope binning metrics

When grouping antibody sequences suspected to target different epitopes, the desirable property of the antibody grouping method is to place antibodies against a single epitope in a single cluster (Robinson et al., 2021; Spoendlin et al., 2023). For this reason, we employed a measure called multiple occupancy consistent clusters members fraction (MOCM), introduced by Spoendlin et al. (Spoendlin et al., 2023). If a non-singleton cluster only consists of antibodies known to target a single epitope, it is counted as a consistent cluster, otherwise it is not.

## Results

### Parameter exploration of the grouping methods on PTx and OVA datasets

**FIGURE 1:**
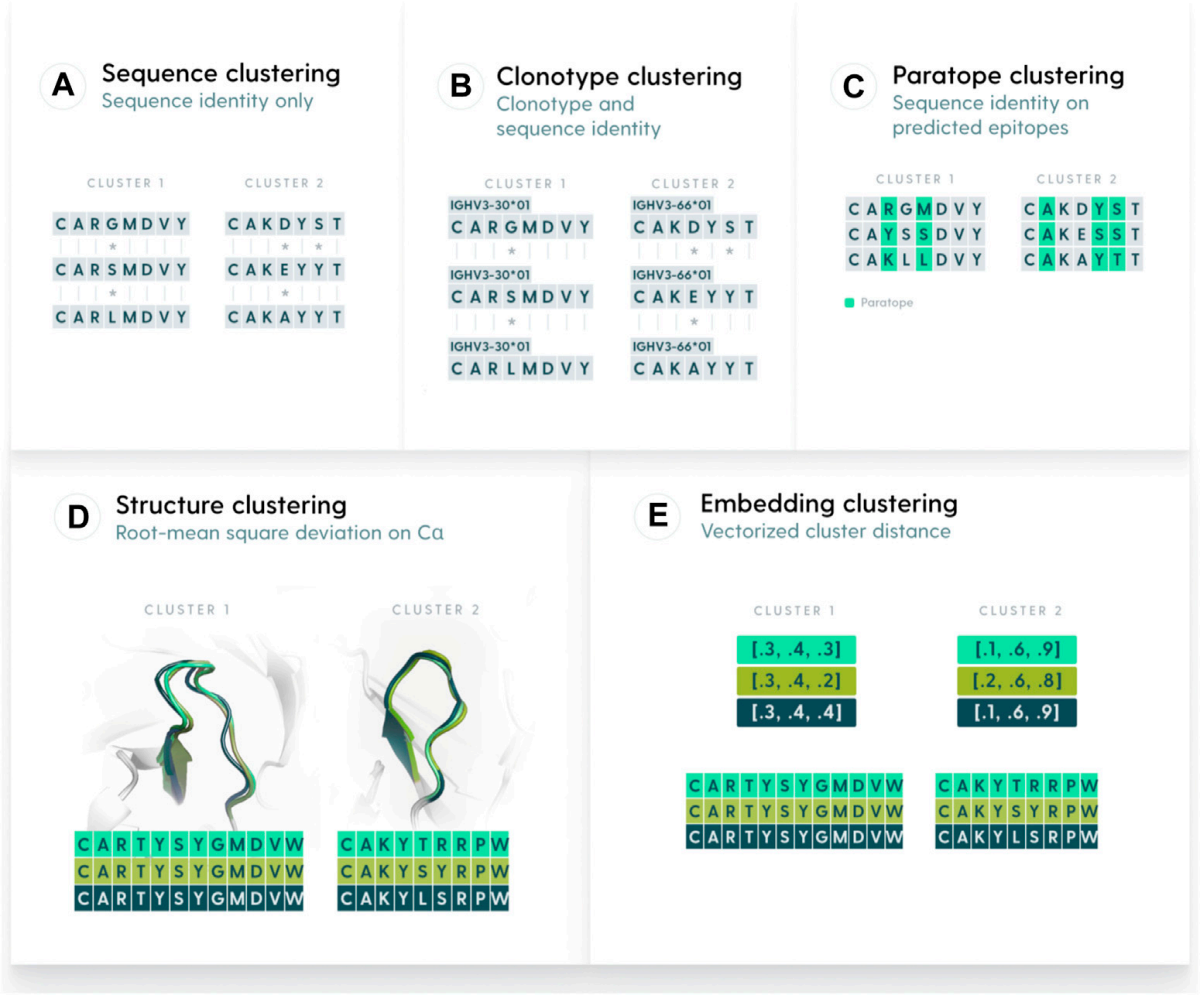
Methods used to group antibody sequences. **(A)**. Sequence-based clustering groups antibodies on the basis of sequence identity. The identity can be taken over the entire variable region or be focused only on certain elements, such as CDR-H3. A variation of sequence clustering, called clonotyping, groups sequences by their assigned genes (e.g., IGHV3-23), CDR-H3 length, and further stratifies on CDR-H3 identity. **(B)**. Clonotype-based clustering. Sequences with the same V or V/J calls are grouped and sequence identity is calculated on length-matched CDR-H3s **(C)**. Paratope-based clustering predicts residues likely to be part of the paratope. The sequence identity is computed only on such residues. **(D)** Structure-based clustering groups antibodies by calculating the Root Mean Square Deviation of their 3D representations. **(E)**. Embedding-based clustering changes the sequence representation into vectors (embeddings) that are efficient representations of sequences trained by a transformer model.

We defined five similarity metrics by which to perform clustering, namely, clonotyping, sequence-based, paratope-based, structure-based, and embedding-based (Figure 1). We benchmarked each of the methods on the OVA and PTx datasets with the aim to characterize their parametrizations and maximum performance. Ideally, a method would have similar parametrization across different datasets, otherwise the behavior between datasets would be unpredictable.

The basic threshold we employed for sequence-based clustering was the sequence identity cutoff. Variations included whether we were stratifying by CDR-H3 length and which region the sequence identity was calculated on (combined CDR-L3+CDR-H3, only CDR-H3, heavy sequence, or light sequence). In both PTx and OVA datasets, stratification by CDR-H3 length does not appear to offer a large benefit in terms of the best F1 (Supplementary Table S1). Stratification appears to increase the bottom interval, in that for both datasets, the bottom F1 is better with stratification rather than without stratification. The best-performing parametrizations for both datasets are distinct - L3+H3 identity and no CDR-H3 stratification for PTx (F1 = .83) and CDR-H3 identity with CDR-H3 length stratification for OVA (F1 = .93). Since the region on which one calculates identity is different, thresholds are not directly comparable. We conclude that in the case of sequence identity, the parametrizations can be largely dataset-dependent.

A facet of sequence clustering that is typically used in antibody grouping is clonotyping. Though there is no set definition of clonotyping, it by and large includes grouping by the assigned genes, and further by CDR-H3 identity (Briney et al., 2016; Briney et al., 2019; Soto et al., 2019; Jones et al., 2020). In principle, sequence-based clustering operates in a similar fashion to clonotyping whence the entire variable region is grouped. Nonetheless, for completeness, we also benchmarked the clonotyping on PTx and OVA datasets with results presented in Table 3; Supplementary Table S2. In clonotyping, we employ two gene-based stratifications, by combination of V-J gene calls and V-only. Sequence identity can be calculated on the entire variable regions, CDR-H3+L3, or CDR-H3 only (which is canonical to clonotyping). Grouping by V-gene only appears to achieve marginally better results, with the best F1 for PTx being 0.80 for the V-J combination and 0.82 for V-only, and both close to 0.9 in the case of OVA. Calculation of identity by CDR-H3+CDR-L3 achieves the best F1 scores on the PTx dataset (F1 = 0.82), but the canonical CDR-H3 only approach works better in lineage annotation of OVA (F1 = 0.9). The best F1 values are comparable between sequence and clonotyping approaches, on PTx 0.83 vs. 0.82 and on OVA 0.93 vs. 0.9 for sequencing and clonotype respectively. Given that in both cases we do not note uniform parametrizations, this further indicates that there might not exist an optimal parametrization, the solution being data-driven.

**TABLE 3:** Best parametrizations.

Dataset	Clustered by	f1	Threshold	cl_res	cl_len	method_specific
PTx	sequence	0.831625	0.772632	l3_h3	none	none
	clonotype	0.822485	0.617368	l3_h3	cdrh3	genes: V
	embedding	0.821229	0.070041	l3_h3	cdrh3	transformer: 768
	paratope	0.801737	0.620000	paratope	cdrh3	none
	structural	0.794521	1.051020	cdrs_all	cdrh3	none
OVA	embedding	0.945114	0.013184	cdrs_all	cdrh3	transformer: 144
	sequence	0.935864	0.648421	cdrh3	cdrh3	none
	clonotype	0.909091	0.648421	cdrh3	cdrh3	genes: V
	paratope	0.906915	0.660000	paratope	cdrh3	none
	structural	0.891583	0.530612	cdrs_all	cdrh3	none

For each of the five clustering methods, we report the best parametrization that was obtained on either the PTx or OVA datasets. “f1” - harmonic mean of the precision and recall, “cl_res” - clustering by residues selected from IMGT regions of sequence, “cl_len” - stratification by length of residues in selected IMGT regions.

The cognate method of clonotyping and sequence clustering is paratope clustering. Here, the sequence identity is calculated solely on the residues that are predicted to be in contact with an arbitrary antigen. The paratope predictions are performed in the absence of the antigen as it was demonstrated to still produce satisfactory results (Liberis et al., 2018), making application to large sets of antibodies with unknown antigen-binding properties possible. Constraining the predictions only to the paratope residues was hypothesized to only focus the grouping on the residues most pertinent to antigen recognition (Richardson et al., 2021). For paratope clustering, we employed a transformer-based paratope predictor (Leem et al., 2022). The only parametrization here was the stratification of CDR-H3 length. Grouping the sequence by the same CDR-H3 length appears to achieve better F1 scores, with 0.80 vs. 0.77 in the case of PTx and 0.9 vs. 0.87 in the case of OVA (Supplementary Table S3; Table 3). The best F1 scores for both datasets are achieved for thresholds 0.62 for PTx and 0.66 for OVA.

Sequence and paratope clustering aim to group antibodies by their antigen-recognizing features. Though sequences and paratope predictions are useful proxies, the antigen-complementarity is ultimately defined by structure. We thus benchmarked the structural clustering. Here, one needs to obtain a 3D model of the variable regions to be grouped. We employed two methods to contrast their speed - NanoNet (Cohen et al., 2022) in its unoptimized form (Kończak et al., 2022) and AbodyBuilder2 (Abanades et al., 2023), the state of the art in antibody modeling. The methods represent two opposites of the spectrum, with NanoNet being very fast (milliseconds per prediction) but performing slightly worse than the slower (seconds per prediction) AbodyBuilder2, which is based on AlphaFold2 (Jumper et al., 2021).

When models are created, RMSD between these is calculated using various schemes (length stratification, constraining to specific regions) to use as a distance measure for clustering. If one were to perform it in an exhaustive manner than for clustering a 1,000 sequence, this starts to be a computational challenge in performing 499,500 pairwise RMSD calculations. We benchmarked an exhaustive method of mTm-Align (Dong et al., 2018b) and greedy algorithm SPACE (Robinson et al., 2021). We found that the SPACE algorithm is not only faster because of its greedy nature but also achieves much better results, so we opted for it in our protocol.

**FIGURE 2:**
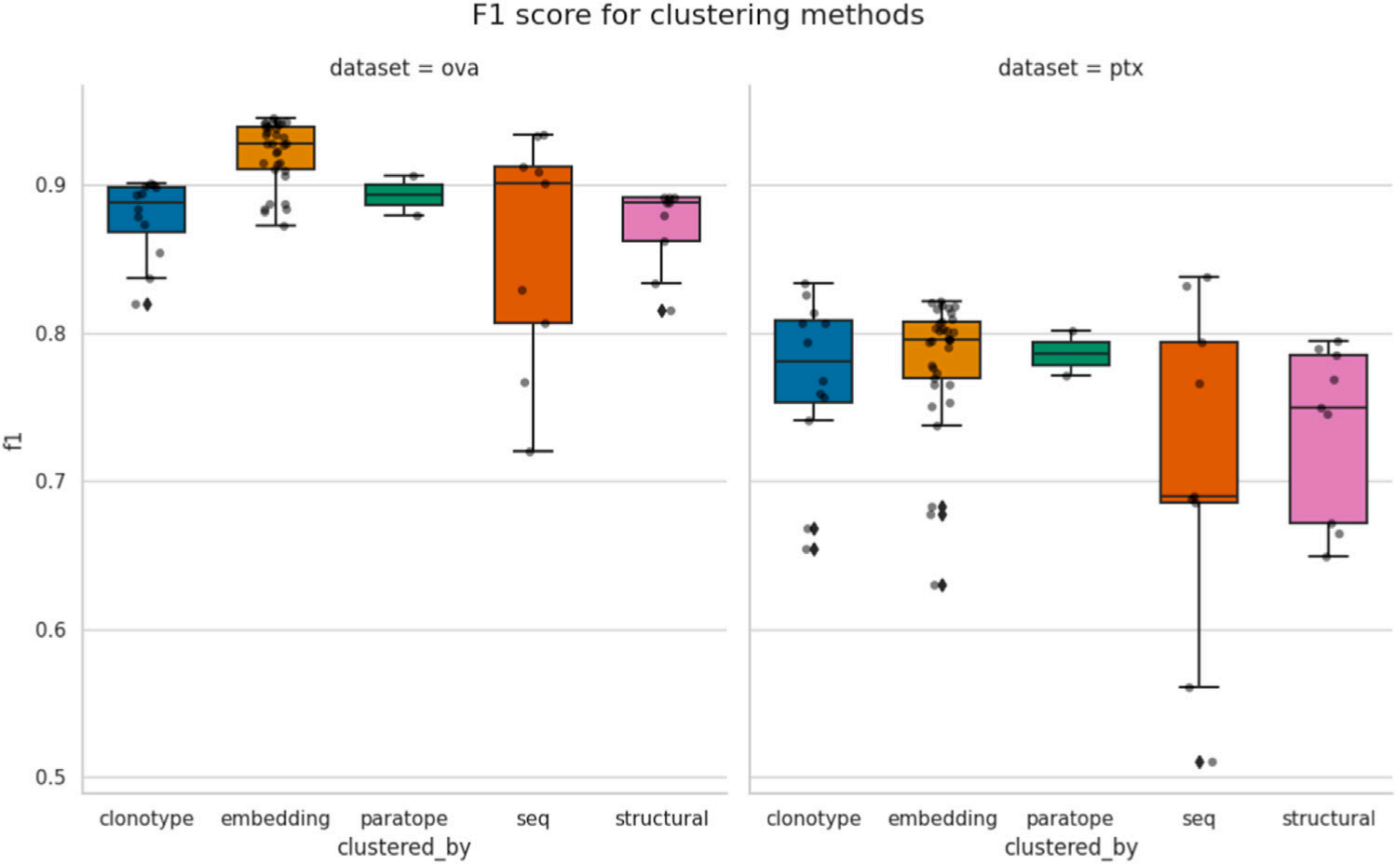
Comparison of best performance achieved by the methods. Each point corresponds to the similarity threshold (e.g., RMSD for structural or sequence identity for sequence) achieving best F1, according to a specific configuration of other parameters, such as length stratification or region where the similarity is calculated. The spread between different methods is given for both the OVA and PTx datasets.“seq” abbreviation means clustering by sequence.

We compared the performance of clustering on PTx between NanoNet and ABodyBuilder2 (ABB2) using the SPACE method. NanoNet achieves marginally worse F1, which is outweighed by its running time (milliseconds to seconds per structure). Therefore, in our protocols, we opted for our implementation of the NanoNet architecture as it achieves similar results but in realistic timelines. AbodyBuilder2 was failing on a number of structures in OVA and reducing the dataset size would be statistically incorrect in comparing all the other similarity methods, so it was left out.

Further to the modeling method, we employed two parameters - length stratification and region where the RMSD is calculated. Stratifying by all CDR lengths and calculating the RMSD on the CDRs produces the best values of F1 (Supplementary Table S4; Table 3). The RMSD cutoff that produced the best results is radically different between the OVA and PTx datasets, with 0.53 and 1.14 respectively, suggesting sensitivity to the method to the input parameters which might not be reproducible across datasets.

The final clustering regime that we explored was based on embeddings from transformers. We trained two versions of transformers - a heavy transformer with an embedding size of 768 (heavy_768) and two paired transformers. The heavy-only transformer was trained on 10 m high-quality heavy chain sequences similar to AntiBERTa and AntiBERTy (Ruffolo et al., 2021; Leem et al., 2022) The paired transformers were trained on the 1.3 m paired NGS dataset (Jaffe et al., 2022). Such models take a long time to train on powerful machines and thus, for the smaller paired dataset, we opted for training a smaller transformer as well for comparison. The clustering was performed on the embeddings from the last layer of the transformer. We picked either the entire CDR-H3, all CDRs, or the entire sequence. The length stratification was performed either on CDR-H3, CDRs, or the entire supplied sequence (only the heavy chain for the heavy transformer).

We plotted the embedding benchmarking results on PTx and OVA in Supplementary Table S5; Table 3. It is evident that, despite the larger number of sequences used for training, the paired transformers achieve better results than the single sequence transformer. For PTx the best F1 for heavy_768 is 0.8 whereas for paired transformers it is 0.82. For OVA the best F1 for heavy_768 is 0.91 whereas for the paired transformers it is 0.94. Within the paired transformers, it is not evident whether calculating the distance on the CDR-H3-CDR-L3 is better than all CDRs as the maximal F1s are comparable. The distance threshold values were taken on the interval 0.001 to 0.2. Therefore, the cutoffs achieving maximal F1s are substantially different between PTx (0.07, 0.07, 0.04) and OVA (0.03, 0.02,0.01). indicating that this method might be very sensitive to input, requiring dataset-specific threshold selection.

### Comparing the performance on the binding on PTx and OVA datasets - All methods achieve comparable performance

Having tested all the parametrizations for individual methods on PTx and OVA datasets, we compared their overall behavior with one another.

In Figure 2, we plot the best F1 scores achieved for all the parametrizations. It is evident that the best F1 performance is dataset-specific, with best values for OVA above 0.9 and for PTx just below 0.8. Our earlier analysis showed that in most cases the thresholds achieving best F1 for each method are dataset-specific as well. Therefore, unsurprisingly, there does not appear to exist a universal parametrization for any single method.

Some methods appear to have a much broader spread of best F1 scores, depending on parametrization. For instance, sequence clustering has the biggest variance within its test results. Clonotyping, which can be thought of as a constraint on sequence clustering, has lower variance. In case of paratope clustering, there were only two parametrizations, which were close to each other and which results in smaller variance. Therefore, care should be taken when interpreting the variance as a different number of parameterizations could have affected it.

Even though we were measuring the maximal F1 a method can achieve on the two datasets, there does not appear to be a method that universally outperforms the others. Conventional sequence-based methods such as sequence clustering and clonotyping are not outperformed by the seemingly more advanced methods. In case of PTx the best F1 are in fact achieved by clonotype and sequence-based clustering. The structural clustering achieved the worst performance. It is difficult to say whether the modeling method is to blame, as crystal structures of the sequences in PTx and OVA datasets are unavailable and two independent modeling algorithms achieved similar performance on both datasets.

**FIGURE 3:**
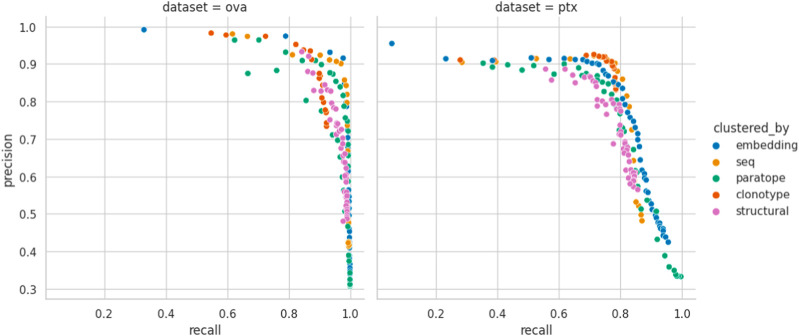
Precision-Recall curve for best parametrizations. Each point corresponds to the precision and recall calculated for the range of similarity thresholds (e.g., RMSD for structural or sequence identity for sequence) for each benchmarked method calculated for the best parametrization. “seq” abbrev. means clustering by sequence. Overlaps between the different clustering methods on OVA and PTx datasets.

Performance appears to be associated with the choice of threshold and, for each method, we picked an optimal parameterization, given in Table 3. For the best parametrization we plotted the Precision-Recall curve to show the behavior of each method, mitigating the impact of imbalanced datasets on visualization, but at varying thresholds (Figure 3). It appears that, regardless of the threshold, the methods are capable of producing very similar results. It was noted previously that paratope and structural clusterings are not in fact better than sequence-based ones (Richardson et al., 2021; Spoendlin et al., 2023). Here we demonstrated this on a wider range of methods and datasets in a constant benchmarking environment.

Structure, paratope, and embedding clusterings do not appear to bring a noticeable advantage over traditional sequence/clonotype-based clustering in terms of better identification of binders. To investigate this, we studied to what extent they produce the same results.

**FIGURE 4:**
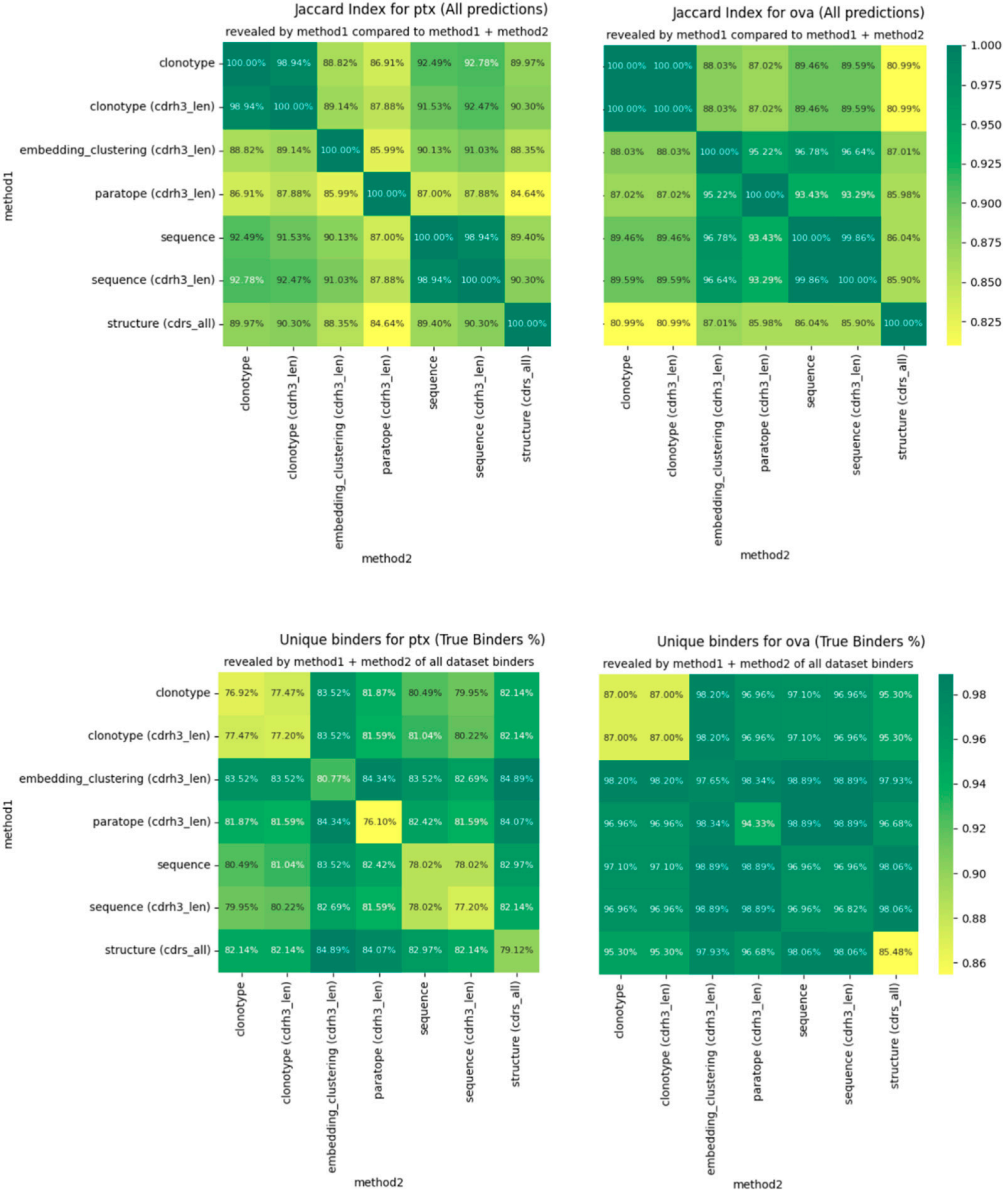
Differences in clustering measured by Jaccard index (top) and percentage of binders identified (bottom).

For any two methods, using their best parametrizations for a specific dataset, we calculated how many sequences were placed in the same cluster by virtue of Jaccard index. If the index is 100%, the two methods produce identical groupings and completely dissimilar ones when it is 0%. We plot the pairwise Jaccard indices in Figure 4 on the PTx and OVA datasets. It is evident that the clusterings do not produce radically different groupings, with Jaccard indices in the region of 85%–90% for most clustering method combinations.

The similarities between clusterings are not reflected perfectly between OVA and PTx datasets. Though CDR-H3 stratified sequence clustering and clonotyping are the most similar methods (92.78%) in PTx, in OVA it is embedding and sequence clustering at 96.78%. Paratope prediction noted the highest dissimilarities in the PTx dataset with the lowest against structural clustering at 84.64%. In the OVA dataset, structure-based clustering had the lowest similarity to the clonotype based methods at 80.99% Jaccard similarity. Therefore, though the methods produce similar results to a large extent, we do not identify anything that would indicate a better complementarity between any pair of these in the form of greater orthogonality.

We also checked to what extent combining the results from two different clusterings would reveal alternative binders. We calculate this as the percent of all the binders that the method returned for the best parametrization, plotted in Figure 4 for PTx and OVA datasets. Here, we note that in each case, combining the results reveals more binders than would be the case if only a single method were used. The result is much more pronounced for PTx than for OVA, but the pattern is visible between the two. Therefore, despite producing similar grouping results, the methods are sufficiently distinct to reveal alternative binders, which we note as the one benefit of other clustering methods over the sequence ones in binder detection.

### Benchmarking the performance of binder calling on blind test sets reveals underlying data and parametrization challenges

Our constraint of the performance on validation sets OVA and PTx reveal that cluster-based binder identification might suffer from parametrization issues. To study the issue further in a realistic discovery scenario, we performed a test on binders from two targets provided by Pure Biologics.

**FIGURE 5:**
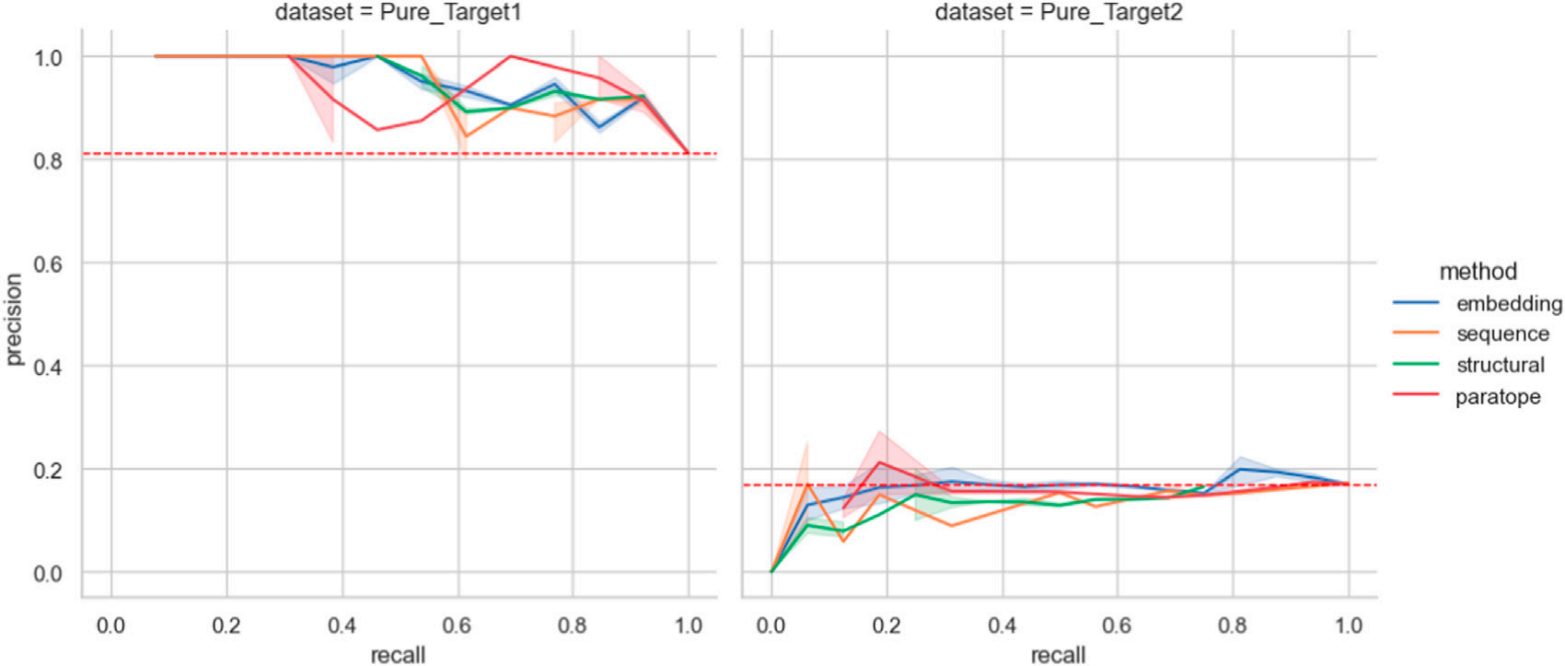
Performance on the Pure Biologics test set. The best parametrization obtained on the training set is used for each clustering algorithm (excluding thresholds). For a full range of threshold values, the precision and recall values are calculated for each grouping method, providing a Precision-Recall curve. Benchmarking grouping method performance on epitope binning dataset.

In both cases, there was an initial set of known binders followed by a blind set. The initial set served as templates of known binders (train set) to cluster the blind test set with these. We first benchmarked the best parametrization on the training datasets with the results given in Table 4. The only method that we could not apply to this dataset was clonotyping, as the number of sequences was too small to provide meaningful groupings. In both cases, embedding clustering achieved the best performance with a comfortable margin away from other methods, as opposed to OVA and PTx datasets (Figure 5), where the differences were not pronounced. In both cases the paired transformers with embedding 768 achieved the best performance. The thresholds were substantially different (0.049 for Pure_ Target1 *versus* 0.033 for Pure_Target2), as was the difference it was calculated on (cl_res).

**TABLE 4:** Performance of top method parameters on training datasets.

		f1	Threshold	cl_res	cl_len	method_specific
dataset	clustered_by					
Pure_Target1	embedding	0.822222	0.049735	l3_h3	none	transformer: 768
	structural	0.764706	1.448980	cdrs_all	cdrh3	none
	paratope	0.750000	0.280000	paratope	none	none
	seq	0.725664	0.865789	heavy	none	none
Pure_Target2	embedding	0.725275	0.013184	cdrs_all	none	transformer: 768
	paratope	0.666667	0.648421	paratope	none	none
	seq	0.666667	0.648421	heavy	none	none
	structural	0.666667	0.530612	cdrh3	cdrh3	none

“f1” - harmonic mean of the precision and recall, “cl_res” - clustering by residues, “cl_res”-clustering by length.

We then employed the training set antibodies as probes on the test sets from the two targets with the Precision-Recall curves for all the methods given in Figure 5. In the case of Pure_Target1, no method achieved better accuracy than a random baseline. In the case of Pure_Target2, the random baseline was already very high as a result of the large number of binders in the dataset, and all the methods were capable of producing values above it. Nonetheless, in an actual drug discovery scenario one requires a particular parametrization that produces the results. We checked the performance of all the parameterizations that achieved best results, both on the Pure training set as well as on PTx and OVA previously (Supplementary Table S6). For both targets, the ‘prior’ parametrizations did not produce results better than random baseline. The baseline F1 for Pure_Target 1 was .89 whereas for Pure_Target2 it was .29.

This blind test set indicates that clustering is not a universal solution to picking good candidates and producing any value is fully dependent on the underlying datasets and parametrization.

Previous results focused on datasets where known binders are to be distinguished on the basis of known ones. This approach is assessed only on its ability to calculate similarity between the probes and the dataset with unknown binders. A cognate problem is to sort the antibodies in a dataset *a priori* based on their antibody-recognition abilities without resorting to the probes. This can be done in epitope binning, where antibodies against known antigen binding sites are to be grouped based on their paratope features alone.

To check the performance of our methods in this scenario, we employed the dataset by Cao et al. (Cao et al., 2023). This dataset consists of 3,501 antibodies against RBD of SARS-COV-2 sorted between 12 epitope groups. The task here is to employ any of our clustering methods and calculate which one performs the best separation of antibodies by their epitopes. The separation is measured using a metric employed from (Spoendlin et al., 2023) - multiple occupancy consistent cluster members (MOCM). According to a particular parametrization of a clustering scheme, we calculate the number of members in clusters with more than one element where antibodies belong to a single epitope. This number is then divided by the number of clustered sequences.

We applied all of our clustering methods to the Cao dataset using the best parametrizations from PTx and OVA datasets as well as optimizing for Cao, with the results in Table 5. As expected, optimization directly on the Cao dataset yielded best results and acted to show the best possible performance of each method. The PTx and OVA dataset parametrizations achieved radically different results, with PTx results being in an acceptable range of the optimal Cao with OVA parametrization heavily underperforming. This shows once again the challenge and data-dependency on clustering any particular dataset.

**TABLE 5:** Performance of epitope binning efficiency measured by fraction of multi-occupancy-consistent clusters members on clustering parameters selected by various datasets.

Clustered by	Threshold source	MOCM fraction	Threshold	cl_res	cl_len	method_specific
space2	Cao	0.275082	1.416667	cdrs_all	cdrh3	none
	PTx	0.253115	1.051020	cdrs_all	cdrh3	none
	OVA	0.089836	0.530612	cdrs_all	cdrh3	none
seq	Cao	0.340656	0.620000	l3_h3	cdrh3	none
	PTx	0.313115	0.772632	l3_h3	none	none
	OVA	0.295082	0.648421	cdrh3	cdrh3	
paratope	Cao	0.414754	0.410000	paratope	cdrh3	none
	PTx	0.362623	0.620000	paratope	cdrh3	none
	OVA	0.322951	0.660000	paratope	cdrh3	none
embedding	Cao	0.297049	0.086286	cdrh_all	cdrh3	transformer: heavy
	PTx	0.345902	0.070041	l3_h3	cdrh3	transformer: 768
	OVA	0.101967	0.013184	cdrs_all	cdrh3	transformer: 144
clonotype	Cao	0.417049	0.524211	l3_h3	none	genes: V
	PTx	0.365902	0.617368	l3_h3	cdrh3	genes: V
	OVA	0.328197	0.648421	cdrh3	cdrh3	genes: V

“MOCM” - multiple occupancy consistent cluster members, “cl_res” - clustering by residues from defined IMGT regions, “cl_len” - stratification by length of residues from defined IMGT regions.

**FIGURE 6:**
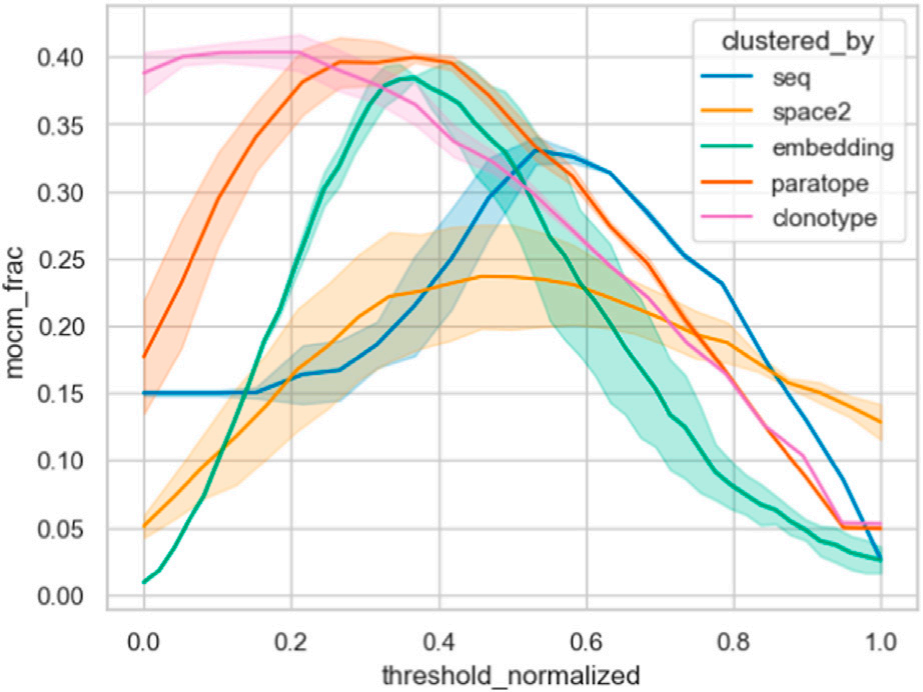
Epitope binning using different clustering methods. The best parametrization was picked for each method, keeping the threshold variable and normalizing it between all methods. We plotted the multiple occupancy consistent clusters, which indicates how many of the final non-singleton clusters consisted only of one epitope. Abbreviation “mocm_frac” means multiple occupancy consistent cluster members fraction.

Furthermore, we contrasted the capacity of the different similarity measures on the Cao dataset by plotting their MOCM performance against normalized thresholds in Figure 6. It is clear that clonotype and paratope clustering outperform all the other methods, with the embeddings in second place. In line with results from Spondelin et al., structural clustering achieved a mediocre performance. Therefore, unlike in previous analyses that relied on the probes, where the results were broadly comparable, in this case we see clear distinction between the performance of the methods in clonotype, paratope, and embeddings generating the best epitope binning splits.

### Web app to study distance distributions

Our analysis indicated that the chief advantage between all the clustering methods is their ability to provide alternative groupings, especially with respect to established methods based on sequence clustering. To facilitate such analyses, we make available a web app that performs the similarity calculation and subsequent grouping using the different schemes introduced here.

The user inputs a set of amino acid sequences in fasta format. The sequences are paired heavy/light sequences without any separation symbol. Users are asked to choose two similarity measures (for instance, structural and embedding) and their parametrizations. In the first step, clustering is performed on the dataset using both methods. Users can then see the results of both clusterings on distance matrix-heatmaps with dendrograms, t-distributed stochastic neighbor embedding (t-SNE) plots of the data side by side for comparison, and a Sankey plot showing the correspondence between clusterings. Since we have shown that the datasets are highly dependent on the choice of threshold for a given dataset, the t-SNE plots, the Sankey plot, and the results of clusterings can be modified using thresholds to provide alternative groupings. The lists resulting from both clusterings are given.

To facilitate selection of candidates using both clusterings, we provide a second step to the clustering that is supposed to enrich the diversity of picks using the novel similarity measures. In the second step, representatives of clusters from one method in step 1 are clustered using the other method. In this fashion, one can reduce redundancy in picks using any clustering (e.g., structural) method, by adding another dimension in the second step (e.g., embedding).

## Discussion

Here we explored the capacity of novel similarity measures made possible by advances in machine learning to group antibody sequences. Machine learning has been applied to many applications in therapeutic antibody discovery and antibody engineering processes. Although a few approaches have been proposed for rapid antibody clustering, still no reasonable consensus or “road map” has been reached in this area. In our studies we checked two main schemes: binder identification via a probe and epitope binning.

In probe-based binder mining, the method is tasked with assessing the similarity of a known binder to those within a sample with unknown specificity. Clearly, if there are binders in the query dataset, but they are not sufficiently similar by any metric, the method would yield no results. Sequence-based similarity is the simplest metric but requires very similar clones in the binding set to work. The machine learning schemes held a promise that they could capture more distant dependences, such as structural composition or even more abstract features encapsulated in the embedding schemes. Our results on the PTx, OVA, and blind test set indicate that, in terms of identifying binders, these methods do not outperform traditional sequence-based schemes.

The opposite is true for the epitope binning exercise. Here one is not dependent on a probe that might not have a close relative in a dataset but rather on the ability to carve the initial dataset in a meaningful way. Here we noted a separation of performance with clonotype, paratope, and embedding schemes surpassing sequence and structure groupings. Therefore, the methods are perhaps more suited in separating a given dataset, as is typically the case in display-type workflow (Erasmus et al., 2023), rather than to perform data-mining experiments.

This is potentially a positive observation, because in realistic drug-discovery exercises one rarely focuses on the scenario of probe-based data mining - the scheme was rather introduced as a way to benchmark bioinformatics methods. However, it could certainly be applied to a biosimilar search. A typical discovery campaign would have produced a large volume of sequences from either immunization or phage display. These then need to be grouped and assessed for similarity to identify enriched clones. Selecting representatives on the basis of sequence identity only runs the risk of picking candidates that are, in reality, not that different from one another (Erasmus et al., 2023).

Our results show that, though there are benefits in using novel clustering methods, they do not offer a universal solution to improving selection campaigns. They should act rather as a tool for diversity exploration that is very much dataset-dependent (Smakaj et al., 2020). By means of rule of thumb of employing the methods, we note that they appear to be well suited to cases where one is faced with a relatively diverse sample. In such a scenario, the task is to provide a sorted list of candidates where diversity is maximized. Having the diversity assessed on several orthogonal levels allows for exclusion of picks that look dissimilar by one metric but are similar according to another. What constitutes a ‘diverse’ sample according to those metrics will also become quantifiable as more discovery-grade datasets reach the public domain on which these can be benchmarked.

Employing public datasets, in line with previous paratope (Richardson et al., 2021), structural (Spoendlin et al., 2023), or embedding (Friedensohn et al., 2020) studies we demonstrated here to what extent the alternative antibody grouping methods provide alternative choices with respect to both sequence-based methods and to one another. To facilitate studying diversity of antibody sequences using different methods, we have created an app (https://clap.naturalantibody.com) that allows one to explore similarity across sequence, embedding, structural, and paratope dimensions of a small sample of antibodies. We are strongly convinced that our analysis of different similarity-based methods sheds light on realistic advantages and provides actionables in terms of better and faster lead candidate selection.

## Data Availability

Publicly available datasets were analyzed in this study. This data can be found here: clap.naturalantibody.com.
