# Supplementary material for: Benchmarking antibody clustering methods using sequence, structural, and machine learning similarity measures for antibody discovery applications
Source: Front Mol Biosci. 2024 Mar 28;11:1352508. doi: 10.3389/fmolb.2024.1352508 (PMC11008471; doi:10.3389/fmolb.2024.1352508)

Supplementary Materials

**Supplementary Table 1.** Sequence clustering accuracy metrics. We use the PTx dataset (363 binding and 749 non-binding paired heavy-light chains) and the OVA dataset (723 binding and 1646 non-binding paired heavy-light chains). Abbreviations mean respectively: “cl_len” - stratification by length of residues selected from IMGT regions, “cl_res” - clustering by residues, “f1” - harmonic mean of the precision and recall.


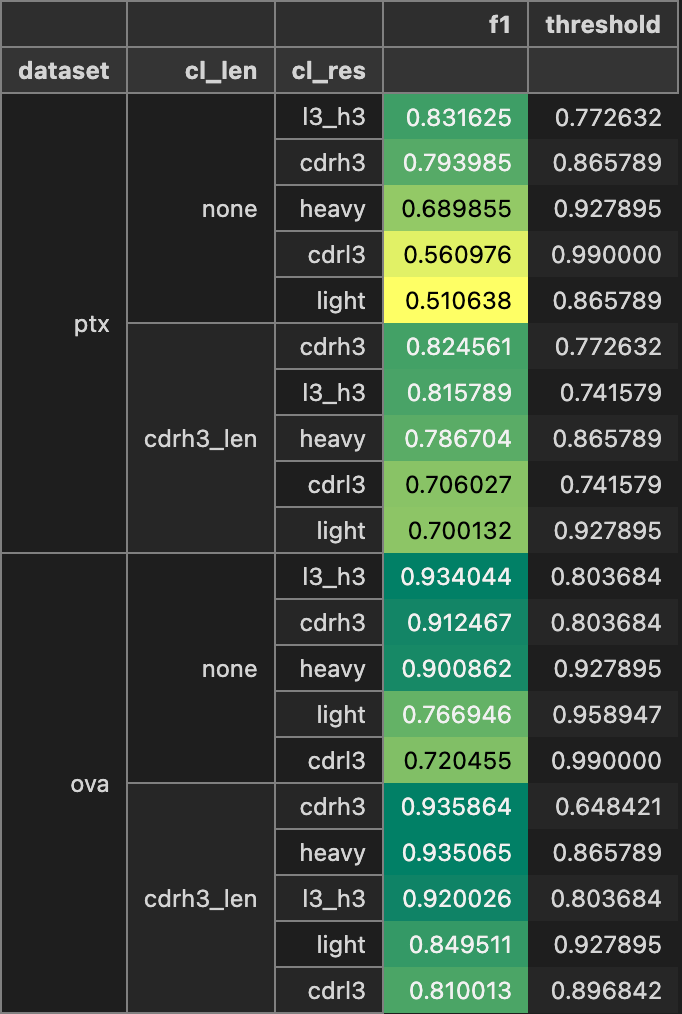


**Supplementary Table 2.** Effect of introducing clonotyping information. We use the PTx dataset (363 binding and 749 non-binding paired heavy-light chains) and the OVA dataset (723 binding and 1646 non-binding paired heavy-light chains). Abbreviations mean respectively: “cl_len” - stratification by length of residues selected from IMGT regions, “cl_res” - clustering by residues, “f1” - harmonic mean of the precision and recall, “cdrh3_len” - CDR-H3 length.


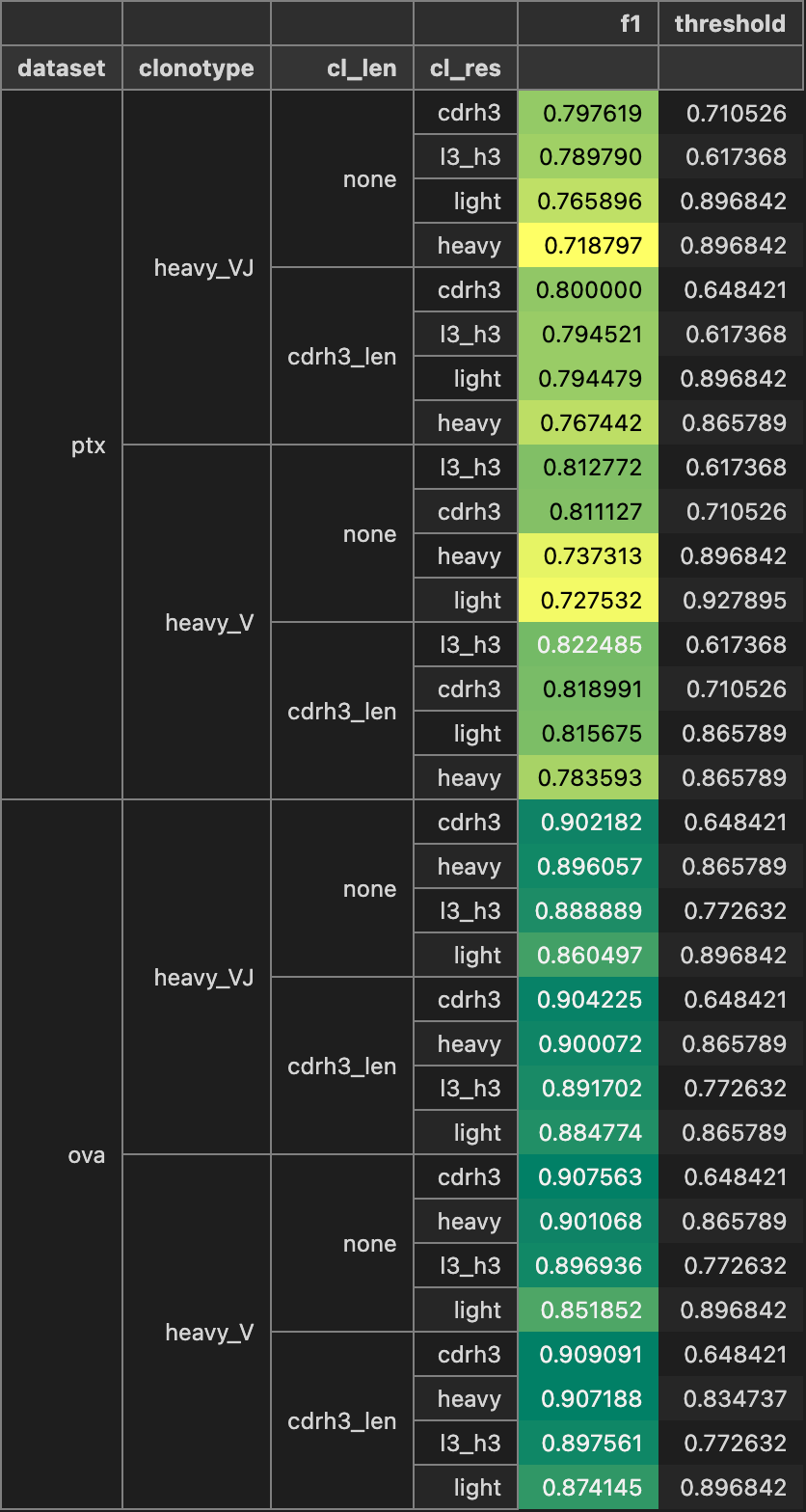


**Supplementary Table 3.** Performance of paratope-based clustering. We use the PTx dataset (363 binding and 749 non-binding paired heavy-light chains) and the OVA dataset (723 binding and 1646 non-binding paired heavy-light chains). Abbreviations mean respectively: “with_cdrh3_len” - stratification by CDR-H3 length, “f1” - harmonic mean of the precision and recall.


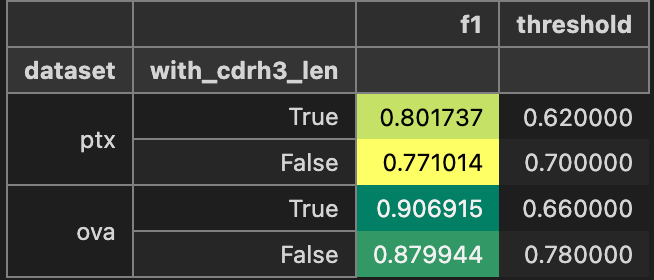


**Supplementary Table 4.** Performance of structure based clustering.We use the PTx dataset (363 binding and 749 non-binding paired heavy-light chains) and the OVA dataset (723 binding and 1646 non-binding paired heavy-light chains). Abbreviations mean respectively: “struct_cource” - source of structural data, “cl_len” - stratification by length of residues selected from IMGT regions, “cl_res” - clustering by residues, “f1” - harmonic mean of the precision and recall.

[Source](https://github.com/NaturalAntibody/structural_clustering/blob/master/notebooks/2.7.1-analysis_clustering_by_structure_space2_CDRs_subset.ipynb)


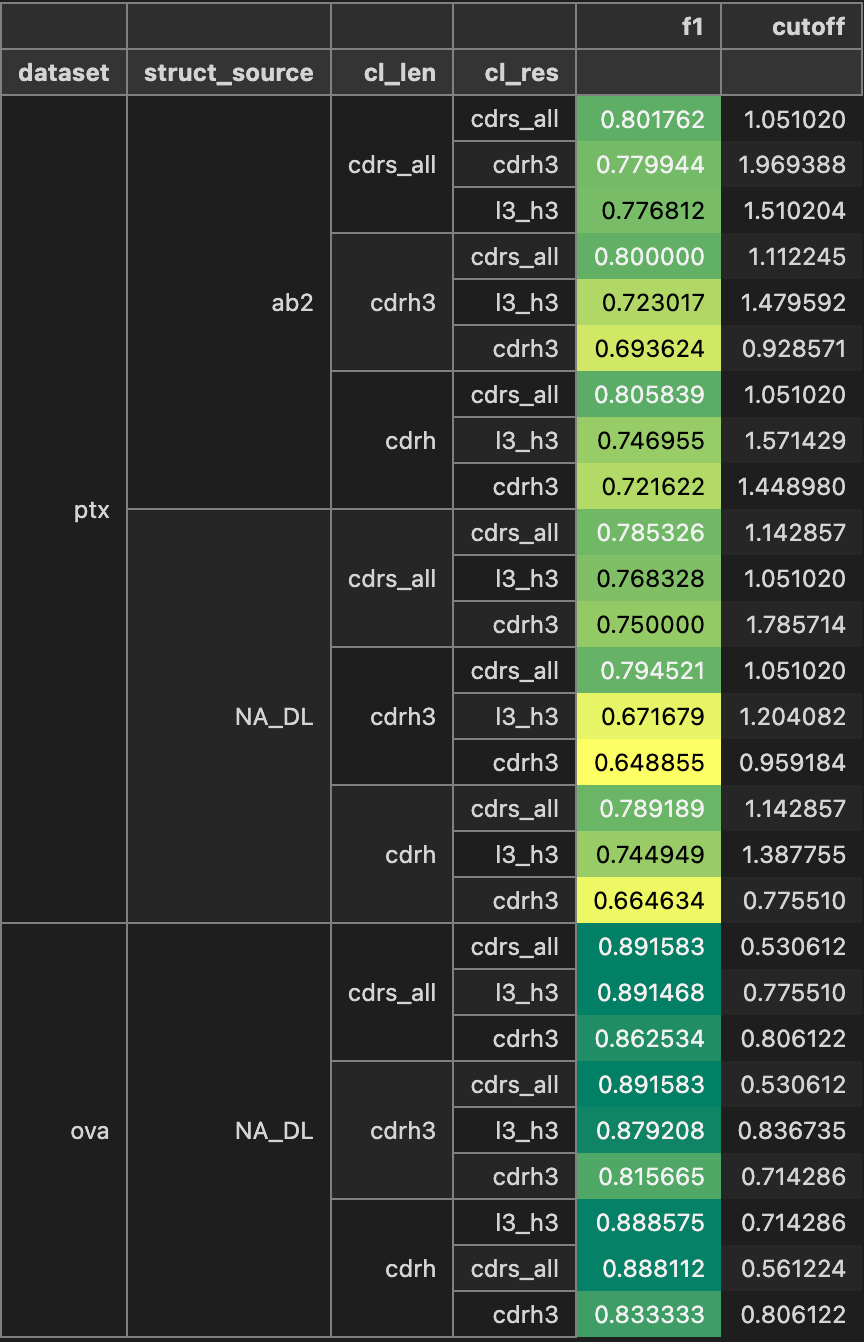


**Supplementary Table 5.** Performance of embedding based clusterings. We use the PTx dataset (363 binding and 749 non-binding paired heavy-light chains) and the OVA dataset (723 binding and 1646 non-binding paired heavy-light chains). Abbreviations mean respectively: “f1” - harmonic mean of the precision and recall.


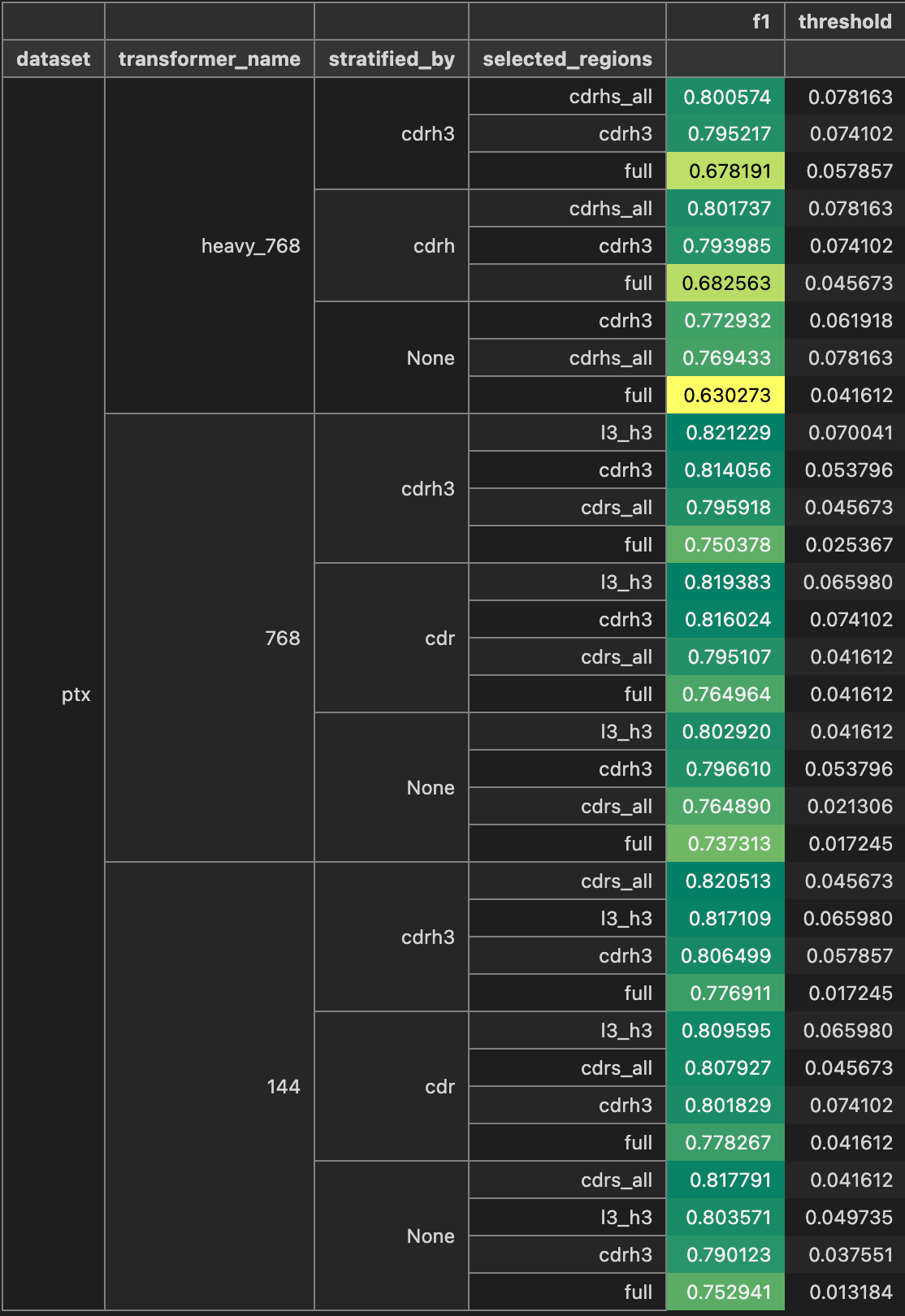


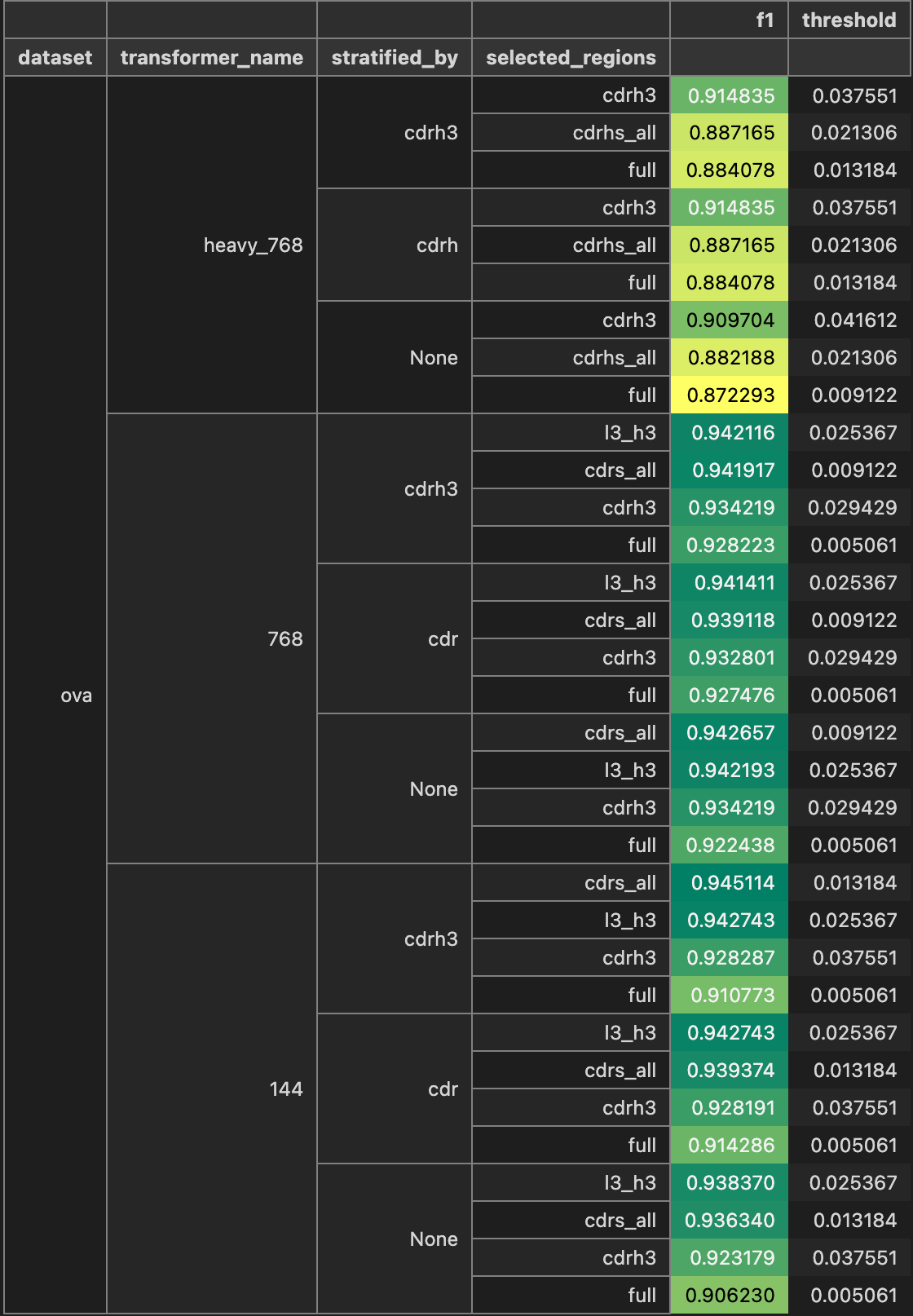


**Table 6.** Performance of different a priori thresholds on target 1 and target 2 test set. We use a binder/non-binder dataset contributed by Pure Biologics for two targets denoted as Pure_Target 1 and Pure_Target2. Abbreviations mean respectively: “f1” - harmonic mean of the precision and recall, “cl_len” - stratification by length of residues selected from IMGT regions, “cl_res” - clustering by residues, “method_specific” - specification of method used.


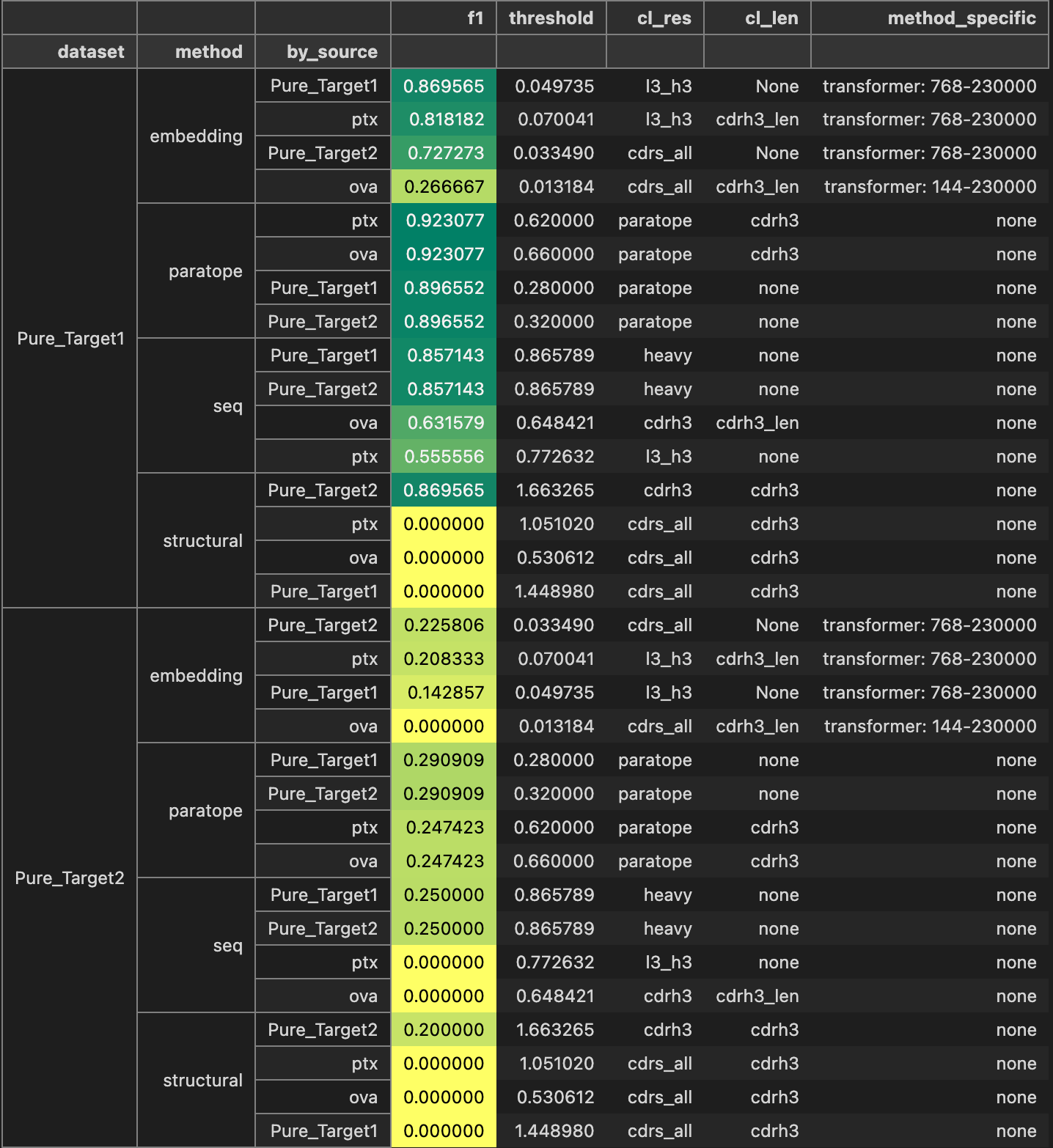


**Supplementary Figure 1.** Sequence diversity across datasets, which was quantified through pairwise sequence identity calculations using Levenshtein Distance applied to residues from selected regions. First image shows diversity in residues selected from all IMGT regions, second image shows diversity in residues selected from CDR IMGT regions from heavy and light chains.


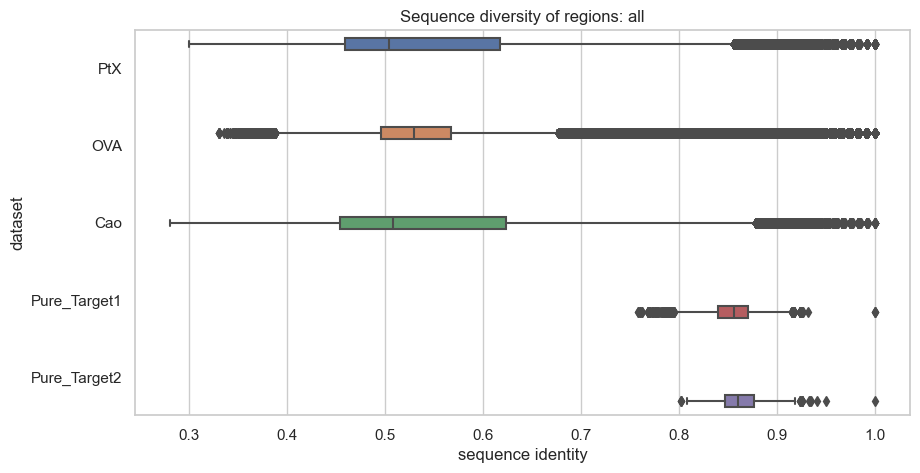


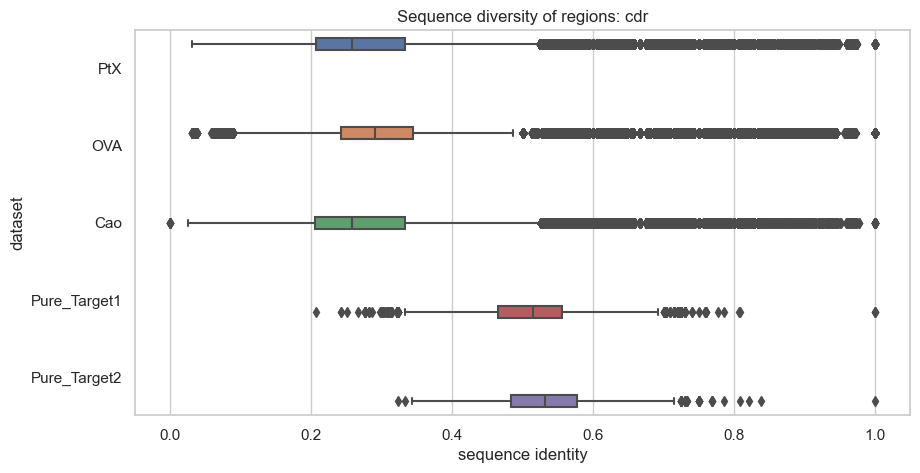

Supplement: Supplementary file 1 [file DataSheet1.docx]
